# The complete plastome of *Bulbophyllum pingnanense* (Orchidaceae: Dendrobiinae)

**DOI:** 10.1080/23802359.2021.1989331

**Published:** 2021-10-14

**Authors:** Sai Zhang, Meng-Yao Zeng, Xu-Yong Gao, Ming-He Li, Shi-Pin Chen

**Affiliations:** a Fujian Colleges and Universities Engineering Research Institute of Conservation and Utilization of Natural Bioresources, College of Forestry, Fujian Agriculture and Forestry University, Fuzhou, China; b Key Laboratory of National Forestry and Grassland Administration for Orchid Conservation and Utilization at College of Landscape Architecture, Fujian Agriculture and Forestry University, Fuzhou, China

**Keywords:** Chinese orchids, chloroplast genome, phylogenetic analysis, Malaxideae

## Abstract

The complete plastid genome of *Bulbophyllum pingnanense*, a critically endangered species, was determined and analyzed in this study. The complete genome was 151,224 bp in length, consisting of a large single-copy region (LSC) of 86,017 bp, a small single-copy region (SSC) of 13,497 bp, and two inverted repeat (IR) regions of 25,855 bp. The genome contained 127 genes, including 81 protein-coding genes, 38 transfer RNA (tRNA) genes, and eight ribosomal RNA (rRNA) genes. Phylogenetic analysis indicated that *B. pingnanense* is sister to *B. inconspicuum*.

## Introduction

*Bulbophyllum*, consisting of more than 1900 species, is one of the two most species-rich genera of Orchidaceae (Pridgeon et al. 2014; Chase et al. 2015). They are widely distributed in tropical Africa, Asia, America, the West Indies, and various islands in the Indian and Pacific Oceans. (Lang and Tsi 1987; Frodin 2004; Pridgeon et al. 2014). *Bulbophyllum pingnanense* Liu et al. (2016, 108), an endemic species of China, falls within the Critically Endangered category (CR, based on criteria B1a + B2a) of the red list guidelines of the World Conservation Union (IUCN 2012). In this study, the complete plastid genome of this species was assembled, annotated and analyzed.

Fresh leaves of *B. pingnanense* were acquired from Pingnan County, Fujian, China (27°01′N, 119°05′E), and voucher specimen was deposited in the herbarium of the Forestry College of Fujian Agriculture and Forestry University (FJFC, Zhang Sai and fafuzs805074688@126.com) under the specimen code J.F. Liu 201312. Total DNA was extracted from fresh leaves using a modified CTAB method of Doyle (1987) and Yang et al. (2014), and quality was assessed by Gel-Electrophoresis.

The GetOrganelle pipeline (Jin et al. 2020) was used to filter the raw sequence reads to get the high-quality plastid-like reads. And then the target-associated reads produced by the former step to get the final FASTA files were assembled using SPAdes within the same pipeline(Bankevich et al. 2012). The assembled plastid genome was annotated based on comparison with *B. inconspicuum* (MN200377) by Geneious Prime^®^ 2020.1.2 (http://www.geneious.com). To detect the phylogenetic relationship of *B. pingnanense* with other Orchidaceae members, additional 24 representative species of Orchidaceae were downloaded from NCBI with three species of Apostasioideae as outgroups. The complete plastomes were aligned with MAFFT (Katoh et al. 2019) using Phylosuite v1.2.2 (Zhang et al. 2020). After nucleotide sequence alignment, the phylogenetic analysis was performed based on the complete plastid genomes using the maximum likelihood (ML), which was conducted on the website CIPRES Science Gateway with RAxML-HPC2 on XSEDE 8.2.10 (Miller et al. 2010). The GTRCAT model was specified for all datasets and 1000 replicates were performed during ML analysis (Stamatakis et al. 2008).

**Figure 1.**
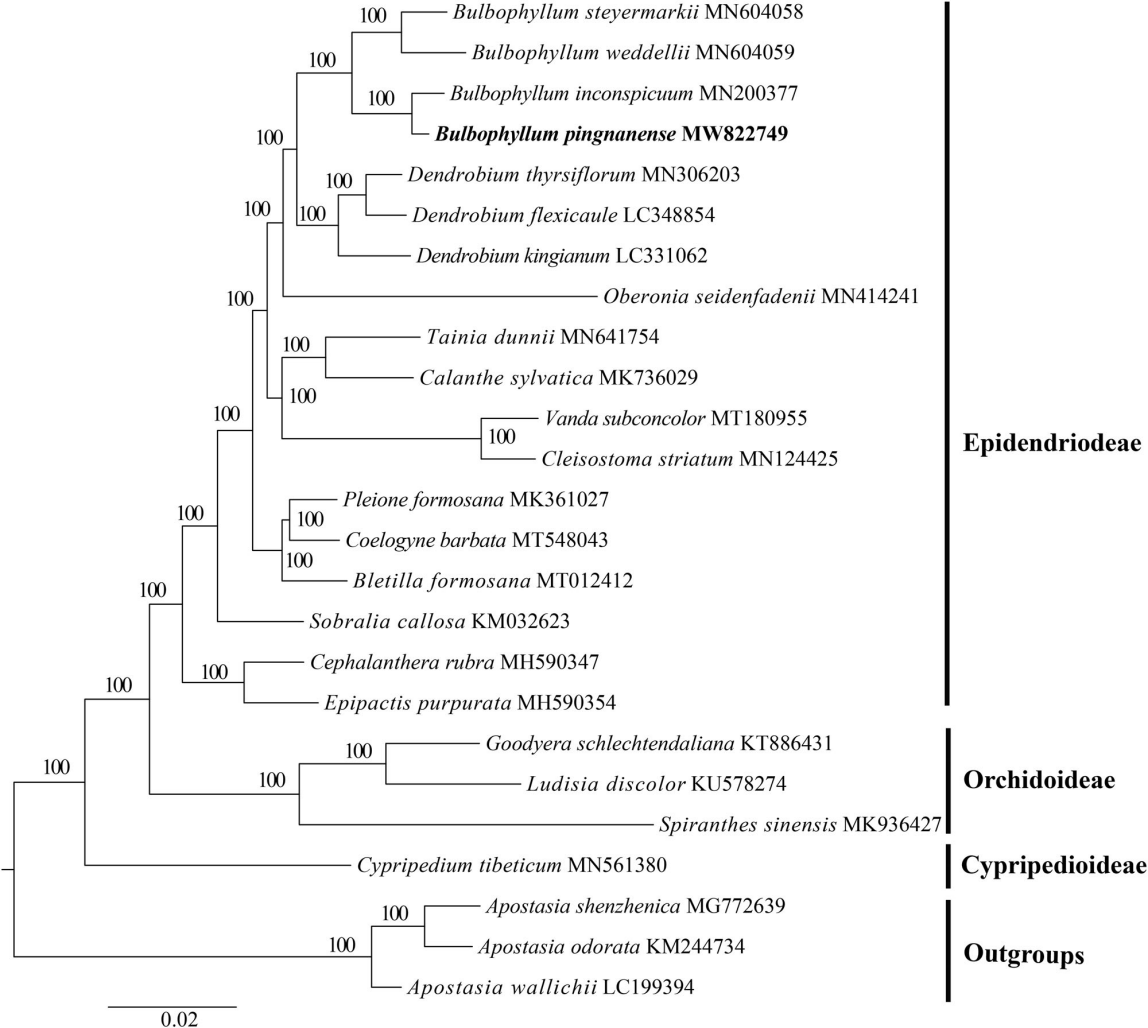
Maximum likelihood analysis based on the 25 representative plastid genome sequences of Orchidaceae with three outgroup species of Apostasioideae. Numbers near the nodes mean bootstrap support value.

A complete circular chloroplast sequence of *B. pingnanense* was 151,224 bp in length (MW822749), which was established to include one large single copy (LSC, 86,017 bp) region, one small single copy (SSC, 13,497 bp) region, and two inverted repeat (IRs, 25,855 bp) regions. The GC content of the complete genome was 37.0% (LSC, 34.4%; SSC, 29.3%; IR, 43.2%) and 127 functional genes were annotated in this plastome, including 81 protein-coding genes, 38 tRNA genes, and 8 rRNA genes. The results of ML phylogenetic tree showed that *B*. *pingnanense* was sister to *B*. *inconspicuum* with a bootstrap value of 100 (Figure 1).

## Data Availability

The data that support the findings of this study are openly available in GenBank of NCBI at https://www.ncbi.nlm.nih.gov, reference number MW822749. The associated BioProject, SRA, and Bio-Sample numbers are PRJNA741590, SRX11326106, and SAMN19871879, respectively.
